# Antihyperglycemic Effect of Methanol Extract of *Syzygium polyanthum* (Wight.) Leaf in Streptozotocin-Induced Diabetic Rats †

**DOI:** 10.3390/nu7095365

**Published:** 2015-09-14

**Authors:** Tri Widyawati, Nor Adlin Yusoff, Mohd Zaini Asmawi, Mariam Ahmad

**Affiliations:** 1 School of Pharmaceutical Sciences, Universiti Sains Malaysia, Minden, Penang 11800, Malaysia; E-Mails: noradlinyusoff@yahoo.com (N.A.Y.); amzaini@usm.my (M.Z.A); mariam@usm.my (M.A.); 2 Department of Pharmacology and Therapeutic, Medical Faculty, University of Sumatera Utara, Medan 20155, Indonesia; 3 Integrative Medicine Cluster, Advanced Medical and Dental Institute, Universiti Sains Malaysia, Penang 13200, Malaysia

**Keywords:** antihyperglycemic, *Syzygium polyanthum* (Wight.), leaf, methanol extract

## Abstract

*Syzygium polyanthum* (*S. polyanthum*), a plant belonging to *Myrtaceae*, is widely used in Indonesian and Malaysian cuisines. Diabetic patients in Indonesia also commonly use it as a traditional medicine. Hence, this study was conducted to investigate the antihyperglycemic effect of the methanol extract (ME) of *S. polyanthum* leaf and its possible mechanisms of action. To test for hypoglycemic activity, ME was administered orally to normal male Sprague Dawley rats after a 12-h fast. To further test for antihyperglycemic activity, the same treatment was administered to glucose-loaded (intraperitoneal glucose tolerance test, IPGTT) and streptozotocin (STZ)-induced diabetic rats, respectively. Hypoglycemic test in normal rats did not show significant reduction in blood glucose levels (BGLs) by the extract. Furthermore, IPGTT conducted on glucose-loaded normal rats also did not show significant reduction of BGLs. However, repeated administration of metformin and three doses of ME (250, 500 and 1000 mg/kg) for six days caused significant reduction of fasting BGLs in STZ-induced diabetic rats. The possible mechanisms of action of *S. polyanthum* antihyperglycemic activity were assessed by measurement of intestinal glucose absorption and glucose uptake by isolated rat abdominal muscle. It was found that the extract not only inhibited glucose absorption from the intestine but also significantly increased glucose uptake in muscle tissue. A preliminary phytochemical qualitative analysis of ME indicated the presence of tannins, glycosides, flavonoids, alkaloids and saponins. Additionally, Gas Chromatography-Mass Spectrometry (GC-MS) analysis detected squalene. In conclusion, *S. polyanthum* methanol leaf extract exerts its antihyperglycemic effect possibly by inhibiting glucose absorption from the intestine and promoting glucose uptake by the muscles.

## 1. Introduction

Diabetes Mellitus (DM) is the number one killer among all chronic diseases in the world [2] and Asians make up more than 60% of the world’s diabetic population [3]. The main symptom of DM is hyperglycemia, which leads to many complications classifiedinto “microvascular” and “macrovascular”. Microvascular complications, such as diabetic nephropathy and diabetic retinopathy, result from damages to the small blood vessels, whereas the macrovascular complications are caused by damages to arteries, leading to coronary artery and periphery artery diseases, and stroke.

Basically, hyperglycemia is the result of relative insulin deficiency, insulin resistance or both [4,5], which renders the cells unable to store glucose. Therefore, the goal of pharmacotherapy is to normalize the blood glucose levels [6]. Currently available conventional antihyperglycemic agents may be divided into a few classes that act to slow glucose absorption from the gut, increasing insulin secretion by β-cells, or increase insulin sensitivity at target tissues. Unfortunately, these oral antidiabetic agents have been reported to precipitate many side effects such as hypoglycemia, weight gain, hepatic dysfunction, metallic taste, abdominal discomfort, *etc*. Therefore, many diabetic patients are inclined to use alternative therapies or traditional herbal medicines, one of which is *Syzygium polyanthum* (Wight).

**Figure 1:**
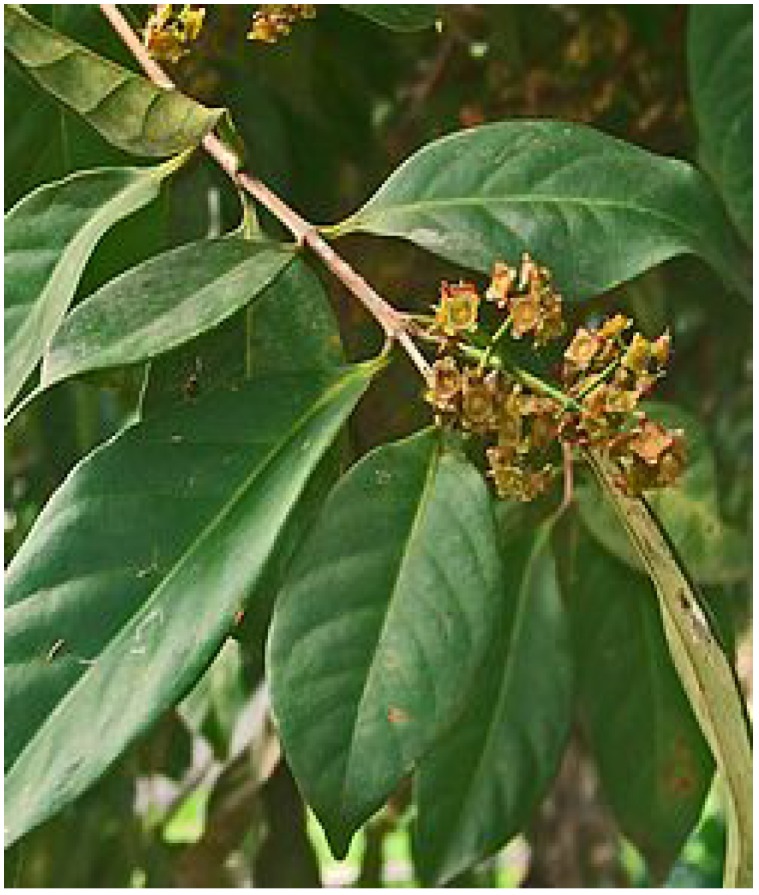
*Syzygium polyanthum* (Wight.).

*Syzygium polyanthum* (*S. polyanthum*), as in Figure 1, is widely used in Indonesian and Malaysian cuisines and is also traditionally used in the treatment of diabetes in Indonesia [7,8]. This plant, belonging to *Myrtaceae* family, is widely distributed throughout Burma (Myanmar), Indo-China, Thailand, Malaysia, and Indonesia (Java, Sumatera, Kalimantan) [7], and has common names, such as *ubar serai*, *meselengan* (Sumatera); *samak*, *kelat samak*, *serah* (Malaysia); *manting* (Jawa), and *Indonesian bay-leaf* or *Indonesia laurel* [7,9]. Phytochemical screening showed that its leaves contained essential oils, tannins, flavonoids, terpenoids and fatty acids [8,9,10]. Interestingly, Patel *et al.* [11] reported that the antidiabetic activity of medicinal plants was attributed to the presence of polyphenols, flavonoids, terpenoids and coumarins.

This study was conducted to investigate the hypoglycemic and antihyperglycemic effects of the methanolic extract of *S. polyanthum* leaf, possible mechanisms of action, and phytochemical content.

## 2. Materials and Methods

### 2.1. Chemicals

Over the course of this study, the following oral antidiabetic drugs were utilized: Glibenclamide (Clamide^®^) 10 mg, Acarbose (Glucobay^®^) 50 mg, and Metformin HCl British Pharmacopoeia (BP) 500 mg, as positive controls for comparison purpose. Additionally, Insulin (NOVO RAPID Flex Pen^®^) 100 IU/mL, and Glucose (R&M Chemicals, Essx, UK) were used. Streptozotocin was purchased from Sigma-Aldrih Chemical Company, St. Louis, MO, USA. Blood glucose levels were determined using Accu-Check Advantage Clinical Glucose meter (Roche diagnostics Co., IN, USA).

### 2.2. Plant Material Collection and Preparation of Extracts

*Syzygium polyanthum* (Wight.) leaves were collected from Titi Kuning, Medan, Indonesia and identified at the School of Biological Sciences, University of Sumatera Utara, Medan, Indonesia (No.13/MEDA/2012). The dried leaves were powdered using a milling machine. The powder, weighing about 1.5 kg, was sequentially extracted by maceration with three solvents to obtain methanol extract (ME). The initial extraction was conducted using petroleum ether (40–60 °C), followed by chloroform and, finally, methanol. The extracts obtained were filtered with Whatman No.1 filter paper and concentrated *in vacuo* by a rotary evaporator (Labortechnik, AG CH-9230, Postfach, Flawil, Switzerland) at reduced pressure.The concentrated extracts were dried in the oven (50 °C) to remove the remaining solvents and the dried extracts were kept in the freezer (−25 °C) until further use in the designated experiments.

### 2.3. Animals

Healthy male Sprague Dawley (SD) rats weighing between 200 and 250 g (*n* = 82) were obtained from the Animal Research and Service Center (ARASC), Universiti Sains Malaysia (USM), after approval by the Animal Ethics Committee of USM Penang, Malaysia (Approval Number: USM/ Animal Ethics Approval/2012/(76)(366)). The animals were acclimatized at room temperature (25–30 °C) for one week; and had access to food (standard laboratory chow, Gold Coin Holdings Sdn. Bhd., Kuala Lumpur, Malaysia) and water *ad libitum* under a 12-h light/12-h dark cycle [12].

### 2.4. Hypoglycemic Test in Normal Rats

Normal male SD rats were divided into six groups (*n* = 6) and fasted overnight (8:00pm–8:00am). The first group, the positive control group, was treated with glibenclamide (10 mg/kg). The second, third, fourth and fifth groups were treated with ME (125 mg/kg, 250 mg/kg, 500 mg/kg, and 1 g/kg, respectively). The sixth group served as the negative control and was treated with normal saline 0.9% (10 mL/kg). All treatments were administered orally using a 16-G oral needle. blood glucose levels (BGLs) were measured before treatment and at the time intervals of 1, 2, 3, 5, and 7 h after each treatment.

### 2.5. Intraperitoneal Glucose Tolerance Test (IPGTT) in Normal Rats

Normal male SD rats were randomly divided into three groups (*n* = 6) and fasted overnight. The first group was treated with normal saline (10 mL/kg). The second and third groups were given metformin (500 mg/kg) and ME (1 g/kg), respectively. All treatments were administered orally. Sixty minutes after drug/extract administration, all the rats were loaded with glucose by intraperitoneal injection (1 g/kg). BGLs were measured from the tail vein before treatment (time 0) and 15, 30, 45, 60, 90, and 120 min after the glucose load [12,13].

### 2.6. Induction of Experimental Diabetes

Streptozotocin solution (prepared in 0.9% NaCl solution, 55 mg/kg) was injected intraperitoneally to overnight-fasted rats. The animals were then observed for 3 × 24 h. After 3 days, the rats’ fasting blood glucose levels were measured. The animals that showed blood glucose levels of 11 mmol/L and above were included in the study.

### 2.7. Antihyperglycemic Test in STZ-Induced Diabetic Rats (SDRs)

Diabetic rats were equally divided into six groups (*n* = 6). The first group was administered with metformin (500 mg/kg). The second, third, fourth and fifth groups were treated with ME (125 mg/kg, 250 mg/kg, 500 mg/kg, and 1 g/kg, respectively). The sixth group was treated with normal saline 0.9% (10 mL/kg). All treatments were administered orally, twice daily, 12 h apart, for 6 days. BGLs were measured before and 6 days after treatment.

### 2.8. Statistical Analysis

All data was expressed as mean ± standard error of the mean (SEM). Mean differences between the groups were statistically analyzed by one-way analysis of variance (ANOVA) at the alpha value of 0.05 followed by Dunnett’stest as a *post hoc* test. Mean differences between pre-treatment and post-treatment were analyzed by paired *t*-test. Differences were considered significant at *p* < 0.05.

### 2.9. Measurement of Glucose Absorption from Rat Everted Jejunal sacs

Glucose absorption from the intestine was determined according to Zurina *et al.* [13] with modifications. Normal rats weighing 200–250 g (*n* = 4) were sacrificed and their abdominal walls dissected. The jejunum, identified as the segment located between 20–25 cm away from the pylorus, was removed and placed in a Tyrode buffer solution (137 mM NaCl, 2.7 mM KCl, 1.8 mM CaCl_2_·2H_2_O, 1.0 mM MgCl_2_, 12 mM NaHCO_3_, 0.2 mM NaH_2_PO_2_ and 5.5 mM glucose) and aerated with Carbogen gas (95% O_2_ and 5% CO_2_). Isolated jejunum were everted using a rod, and cut into 5-cm length segments. One end was tied tightly to form a sac, while the other end was tied loosely with a thread. Each sac was filled with 1 mL of Tyrode’s solution by slipping a blunt needle attached to a syringe through the loose end and tighten the knot as the needle was being withdrawn. Each sac was immersed in a solution containing 1 mg/mL acarbose and/or ME; and one end of the thread was allowed to hang over the opening of the tube. Samples were dissolved in distilled water. All of the tubes were then placed in a water bath (37 °C) and incubated for 1 h. The sacs were removed from the test tubes by pulling on the thread using a forceps after incubation. Three milliliters of the peridochrome-glucose reagent were pipetted into each test tube. Next, 30 μL of each test solution were added into the respective tube. The mixtures were incubated for 20 min in a water bath at 37 °C. Glucose concentration was determined by using a Stat Fax machine 1937 (Awareness Technology Inc., FL, USA).

Glucose absorption was calculated using the following formula:

(1) Glucose absorption (mg_glucose_/dL/g_tissue_) = (Glucose concentration before incubation − Glucose concentration after absorption)/weight of the jejunum segment

### 2.10. Measurement of Glucose Uptake by Isolated Rat Abdominal Muscle

Glucose uptake by isolated rat abdominal muscle was measured according to Gray and Flatt [13,14] with modifications. Normal SD rats (200–250 g) (*n* = 6) were sacrificed and skinned to expose the abdominal muscle. The abdominal muscles were then excised without the rectus abdominus and were cut into small squares (90–150 mg per muscle) and transferred into Kreb’s-Ringer bicarbonate buffer solution (KRB; 18 mM NaCl, 5 mM KCl, 2.0 mM KH_2_PO_4_, 1.2 mM MgSO_4_, 25 mM NaHCO_3_, and 1.28 mM CaCl_2_) in the presence of 95% O_2_ and 5% CO_2_ at 37°C. The muscle segments were then placed in 1.5 mL eppendorf tubes containing KRB solution and were aerated for 10 min to allow them to acclimatize. After acclimitization, the KRB solution was changed with a fresh KRB solution containing 11.1 mmol/L glucose. The muscles were then aerated for 5 min in the presence of test substances with/without the presence of insulin. Insulin stock, obtained at the concentration of 100 IU/mL, was utilized. A 50 IU solution was prepared by taking 500 μLinsulin at 100 IU/mLand adding 500 μL KRB solution. In order to attain insulin at the final concentration of 1 IU/mL in 1 mL of KRB solution to run the experiment using 1.5 mL eppendorf tubes, the calculation was made using the following formula: (M1V1_before dilutio^*n*^_ = M2V2_after dilution_); hence, V1 = ((1 IU/mL × 1 mL)/(50 IU/mL)) = 0.02 mL = 20 μL of insulin at 50 IU/mL. Likewise, 20 μL of the test substances, prepared at 50 mg/mL, were added to the respective tubes. Thus, 960 μLof KRB solution made the volume up to 1 mL. Tubes without insulin saw a similar calculation except that insulin was not added; hence, 20 μL of test substances at 50 mg/mL + 980 μL of KRB solution made up the final volume to 1 mL. Following the 5-min aeration, the tubes were incubated for 30 min at 37 °C in a water bath. To determine glucose uptake, 3 mL of Peridochrome-glucose reagent were pipetted into clean test tubes, into which 30 μL of each test supernatant were added. Next, the mixtures were incubated for 20 min in a water bath at 37 °C; and glucose uptake by the muscles was measured by using a Stat Fax machine 1937 (Awareness Technology Inc., FL, USA).

Calculation:

(2) Glucose uptake (mmol_glucose_/dL/g_tissue_) = ((amount of glucose before incubation (mmol/dL)− amount of glucose after incubation (mmol/dL))/weight of muscle segment (g))

### 2.11. Phytochemical Identification

Phytochemical qualitative analysis was performed using the general method for phytochemical screening of tannins, glycosides, flavonoids, alkaloids and saponins with modification [15,16].

#### 2.11.1. Identification of Tannins

Half a gram of extract was stirred with 10 mL of distilled water and filtered. Four drops of a 1% ferric chloride solution were added to 2 mL of the filtrate. Blue-black, green or blue-green precipitate indicated the presence of tannins.

#### 2.11.2. Identification of Glycosides

Half a gram of extract was dissolved in 1 mL of distilled water. Aqueous NaOH 2M was added to the solution and the resulting yellow coloration indicated the presence of glycosides.

#### 2.11.3. Identification of Flavonoids (Ferric Chloride Test)

Half a gram of extract was boiled with 3 mL of distilled water. The solution was then filtered; and 2 mL of the filtrate were mixed with 5 drops of a 10% ferric chloride solution. The color change to green-blue or violet indicated the presence of the phenolic hydroxyl group.

#### 2.11.4. Identification of Alkaloids (Dragendorff’s Test)

Approximately 0.2 g of extract were stirred with 5 mL of 1% hydrochloric acid (HCl) in a water bath. The solution was filtered; and 1 mL of the filtrate was added to a test tube. Next, Dragendorff’s Reagent was added to the filtrate. The presence of an orange color signaled the presence of alkaloids.

#### 2.11.5. Identification of Saponins

Approximately 0.2 g of extract were dissolved in 5 mL of distilled water and the mixture was heated until boiling. The solution was then cooled and filtered. Next, 3mL of distilled water wasadded to the filtrate and the tube was shaken vigorously. Formation offrothindicatedthe presence of saponins.

### 2.12. Identification by Gas Chromatography-Mass Spectrometry (GC-MS)

ME was dissolved in methanol (1 mg/mL). So far, the literature has not shown any compound with antidiabetic activity to have been reported in *S.polyanthum.* Therefore, our samples were analyzed using GC-MS. Consequently, squalene was found in ME and was then purchased as a reference compound (Squalene (synonym:2,6,10,15,19,23-Hexamethyl-2,6,10,14,18,22-tetracosahexaene((CH_3_)_2_C(=CHCH_2_CH_2_C(CH_3_))_2_=CHCH_2_-)_2_; molecular weight 410.72, >98% liquid) from SIGMA. It was diluted in methanol at a 1 mg/mL concentration.GC-MS analysis was conducted under the following conditions: column used was HP-5MS; initial oven temperature was 70 °C (2 min) and was raised to 280 °C at the rate of 20 °C per min; final time: 20 min; total run time: 32.50 min; flow: 1.2 mL/min;splitless. One microliter of ME solution was injected into the GC apparatus. Mass spectrometry conditions: scan parameter for low mass 35.0 and high mass 650.0; MS Quad: 150 °C; MS Source: 230 °C.The resulting mass spectrum was analyzed using the database of the National Institute of Standards and Technology (NIST 02). Squalene was considered a match with the match quality being >90%, which indicated good similarity with the spectrum of squalene in the library. Confirmation of the result was conducted with ion fractionation.

## 3. Results

### 3.1. Hypoglycemic Test and IPGTT in Normal Rats

**Figure 2:**
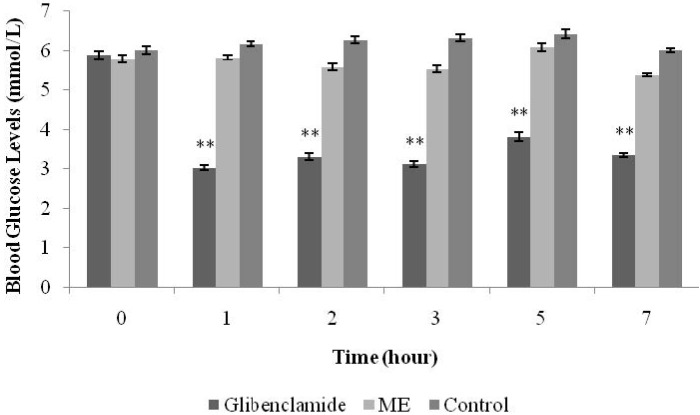
Effects of the methanolic extract (ME) of *S. polyanthum* leaf, and glibenclamide on blood glucose levels of normal rats. Values are expressed as mean ± standard error of the mean (SEM) (*n* = 6); ** *p* < 0.01 compared with the control group.

Figure 2 shows the effects of glibenclamide and ME on BGLs. Oral treatment with ME (1 g/kg) did not significantly alter BGLs of normal rats as compared to the control group during the 7-h observation period. However, BGLs in glibenclamide (*p* < 0.01)-treated rats were significantly reduced from the 1st until the 7th h after the oral treatment.

**Figure 3:**
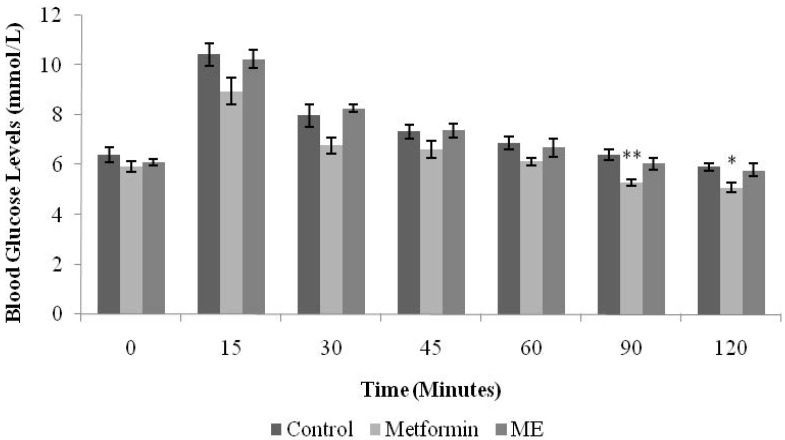
Effects of the methanolic extract (ME) of *S. polyanthum* leaf on blood glucose levels following intra-peritoneal glucose loading in normal rats. Values are expressed as mean ± standard error of the mean (SEM) (*n* = 6); * *p* < 0.05, ** *p* < 0.01 compared with the control group.

Figure 3 shows the IPGTT test in normal rats. The increase in BGLs upon glucose loading was not significantly altered by oral treatment with ME (1 g/kg), within 120 min after glucose loading. However, BGLs in metformin-treated group were significantly decreased at 90 and 120 min (*p* < 0.01, *p* < 0.05, respectively).

### 3.2. Antihyperglycemic Test in STZ-Induced Diabetic Rats (SDRs)

**Figure 4:**
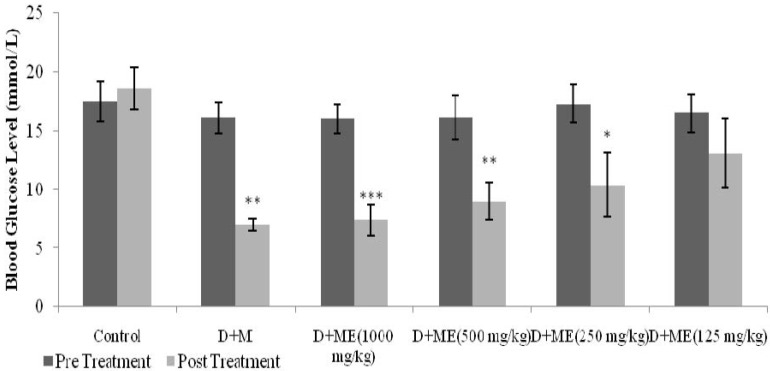
The effects of oral administration twice daily of 125 mg/kg, 250 mg/kg, 500 mg/kg, 1000 mg/kg of the methanolic extract (ME) of *S. polyanthum* leaf and metformin (M) 500 mg/kg on blood glucose levels in streptozotocin-induced diabetic rats. Values were expressed as mean ± standard error of the mean (SEM) (*n* = 6); mean differences between pre-treatment and post-treatment were analyzed by paired t-test, * *p* < 0.05; ** *p* < 0.01; *** *p*< 0.001.

Figure 4 depicts the antihyperglycemic effects of ME and metformin on BGLs after six days of oral administration twice daily. As shown below, oral administration of ME at a dose of 125 mg/kg did not manage to significantly decrease SDRs’ fasting BGLs. However, doses of 250 mg/kg, 500 mg/kg and 1000 mg/kg of ME significantly (*p* < 0.05, *p* < 0.01*, p* < 0.001) and dose-dependently reduced their fasting BGLs. Likewise, metformin as a positive control caused a significant reduction (*p* < 0.01) in the BGLs.

### 3.3. Glucose Absorption from the Intestine

**Figure 5:**
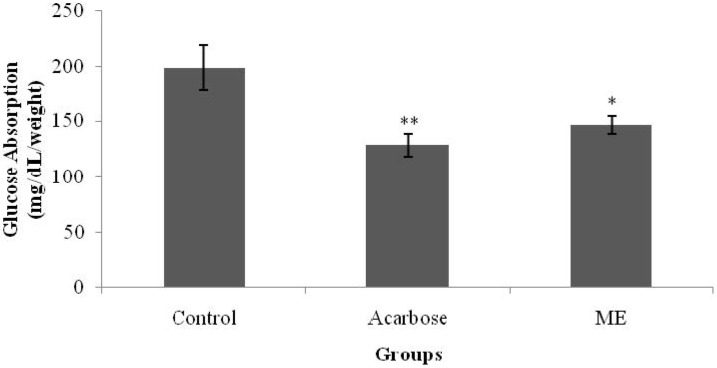
The effect of methanolic extract (ME) of *S. polyanthum* on glucose absorption of everted rat jejunum. Values are expressed as mean ± standard error of the mean (SEM) (*n* = 5); * *p* < 0.05, ** *p* < 0.01 compared with control group.

Figure 5 shows the *in vitro* effects of ME and acarbose on intestinal glucose absorption. As shown below, in the absence of acarbose and ME, the amount of absorbed glucose was 198.2 mg/dL/g tissue weight. Compared with the control, the presence of acarbose, however, caused a significant reduction in glucose absorption (*p* < 0.01), decreasing the value to 128 mg/dL/g tissue weight. ME showed a similar hopeful result as the amount of glucose absorbed was significantly reduced to 146.5 mg/dL/g tissue weight, as compared with the control.

### 3.4. Glucose Uptake by Isolated Rat Abdominal Muscle

**Figure 6:**
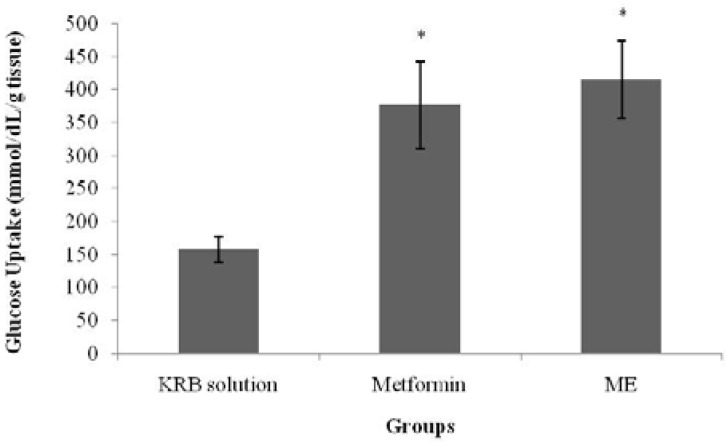
The effect of the methanolic extract (ME) of *S. polyanthum* and metformin on glucose uptake by isolated rat abdominal muscle in the absence of insulin. Values are expressed as mean ± standard error of the mean (SEM) (*n* = 5); * *p* < 0.05, compared with the control group (KRB solution).

**Figure 7:**
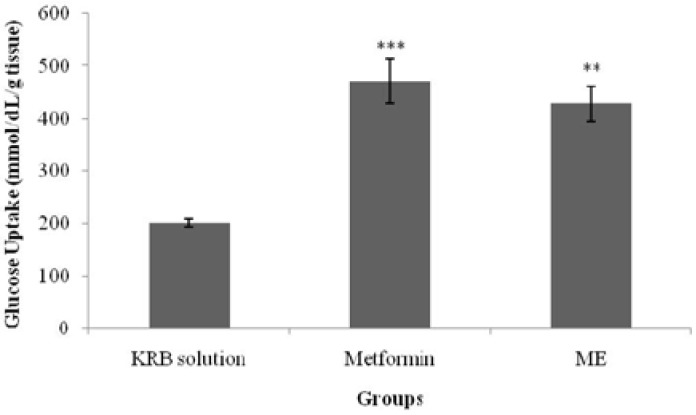
Effects of the methanolic extract (ME) of *S. polyanthum* and metformin on glucose uptake by isolated rat abdominal muscle in the presence of insulin. Values are expressed as mean ± standard error of the mean (SEM) (*n* = 5); **p < 0.01, *** *p* < 0.001 compared with the control group (KRB solution).

Figure 6 and Figure 7 show glucose uptake by isolated rat abdominal muscle in the presence and absence of insulin. In normal isolated rat abdominal muscle, with the absence of insulin, a significant increase (*p* < 0.05) in glucose uptake was observed in the metformin-treated group (377.7 ± 67.8 mmol/dL/g tissue weight) when compared with the control group (158.1 ± 20.5 mmol/dL/g tissue weight). Similarly, treatment with ME also elicited a significant increase in glucose uptake (*p* < 0.05) (416.2 ± 59.9 mmol/dL/g tissue weight) when compared with the control in the absence of insulin. The presence of insulin in the KRB solution significantly increased glucose uptake in both the metformin- and ME-treated groups (471.9 ± 42.6 mmol/dL/g tissue weight (*p* < 0.001) and 428.6 ± 33.1 mmol/dL/g tissue weight (*p* < 0.01), respectively) when compared with the control and insulin treatment alone.

### 3.5. Phytochemical Identification

**Figure 8:**
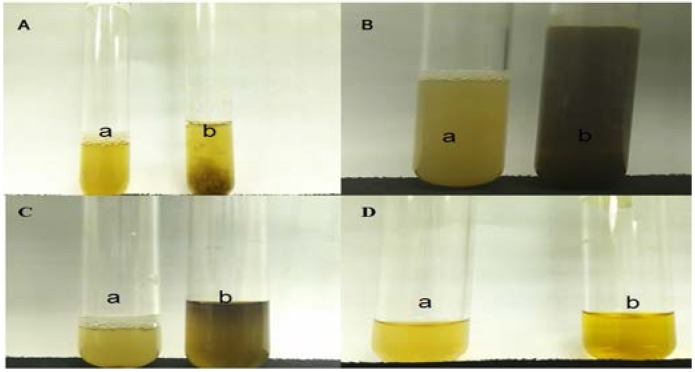
Phytochemical qualitative analyses of *S. polyanthum* leaf methanolic extract (ME): (**A**) Green-blue precipitate (b), indicating the presence of tannins (a: before; b: after ferric chloride reagent (Fe_2_Cl_3_) addition). (**B**) A light yellow color (b), indicating the presence of glycosides (a: before; b: after sodium hydroxide (aq) addition). (**C**) Green blue coloration (b), indicating the presence of flavonoids (a: before; b: after sulfuric acid (H_2_SO_4_) addition). (**D**) An orange color (b), indicating the presence of alkaloids (a: before; b: after Dragendorff’s reagent addition).

Figure 8 depicts the phytochemical qualitative analysis aimed at detecting the presence of tannins, glycosides, flavonoids and alkaloids in ME. Positive results were shown for the tannins, glycosides, flavonoids and alkaloids tests.

### 3.6. GC-MS Analysis

**Figure 9:**
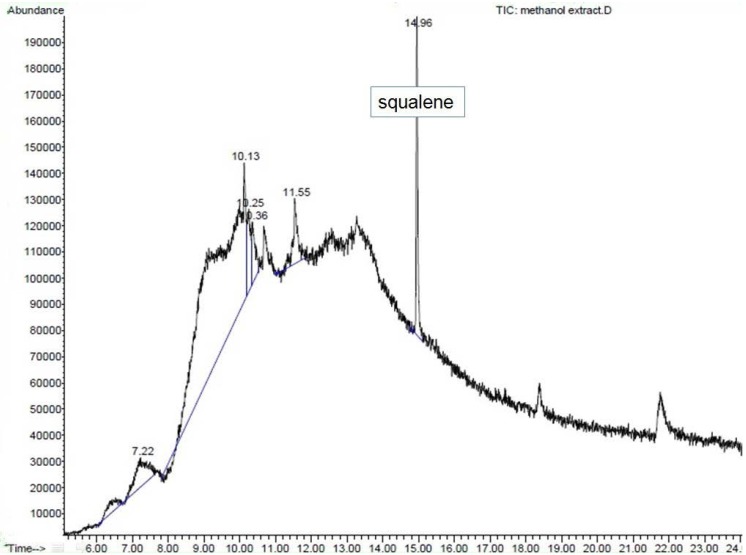
Gas Chromatography–Mass Spectrometry (GC-MS) chromatogram of methanol extract (ME) of *S. polyanthum* leaf with squalene as standard.

GC-MS analysis of the methanolic extract of *S. polyanthum* leaf showed that it consisted of many constituents (data not shown) that may contribute to its medicinal activity. The analysis was based on the quality of matching with the library (>90%). Hence, only one compound was identified with high certainty as squalene (Figure 9).

## 4. Discussions

The present study was conducted in normal and diabetic rats to investigate the hypoglycemic and antihyperglycemic effects of *S.polyanthum* (Wight) leaf methanolic extract. Preliminary studies were conducted in normal rats. For the hypoglycemic test, an antidiabetic standard drug of the sulphonylureas class (glibenclamide) was used as the positive control, whereas a biguanide (metformin) was chosen for the IPGTT. The hypoglycemic test showed glibenclamide to reduce BGLs from the first hour up until the seventh hour after oral administration. Glibenclamide acts by increasing insulin secretion from the pancreatic beta cells by closing the adenosine triphosphate-sensitive potassium channels, depolarizing beta cell plasma membranes, and increasingintracellular calcium concentrations—thus stimulating insulin release. These actions are responsible for the effectiveness of glibenclamide in decreasing both fasting blood glucose levels and postprandial hyperglycaemia [16]. Treatment with a single dose of ME did not affect the BGLs of normal fasting rats. Hypoglycemia is a condition characterized by low blood glucose levels, levels below 3.8 mmol/L. Glibenclamide at the present study showed the ability to reduce BGLs to be within the hypoglycemic range (5.9 ± 0.11 mmol/L to 3.4 ± 0.05 mmol/L), which validated its activity as a hypoglycemic agent.

InIPGTT, only metformin inhibited the rise of BGLs significantly at the 90 and 120 min intervals (*p* < 0.01, *p* < 0.05, respectively). Metformin, a biguanide, acts as an insulin sensitizer *i.e.*, its blood glucose lowering action does not depend on the presence of functioning pancreatic β-cells [17,18]. Patients with type 2 diabetes show considerably less fasting hyperglycemia as well as lower postprandial hyperglycemia after biguanide intake. Therefore, biguanides are appropriately termed the “euglycemic” agents [18]. Since ME showed insignificant effect in IPGTT in the normal rats, we conducted the test in streptozotocin-induced diabetic rats.

Streptozotocin (STZ) is an antibiotic [19] that is naturally synthesized by *Streptomycete sachromogenes* and is used in the laboratory to induce both type 1 and type 2 DM in animals [20]. STZ causes pancreatic beta cell destruction, accompanied by hyperglycemia and a reduction of blood insulin levels [20,21]. In STZ-induced diabetic rats, an antihyperglycemic test with repeated daily dosing for six days showed that both metformin- and ME-treated groups had significantly reduced BGLs, as compared to the control. The present study depicted that different doses of ME reduced BGLs in a dose-dependent manner. Administration of different doses also showed that the minimum dose of ME that still possessed antihyperglycemic activity was 250 mg/kg.

The present study further investigated two possible antidiabetic mechanisms of action of *S.* *polyanthum* ME *in vitro*. The averted jejunal sac method of Wilson and Wiseman (1954), with modifications, was used to investigate the effect of ME on glucose absorption in the intestine. Glucose uptake by the muscles was examined using rat isolated abdominal muscles according to Gray and Flat (1998) with some modifications [21].

Blood glucose levels are maintained within a narrow range by homeostatic mechanisms [5]. Dietary complex carbohydrates are broken down to simple sugars in the gastrointestinal tract by the action of glucosidases [6]. Only monosaccharides, such as glucose and fructose, can be transported out of the intestinal lumen and into the bloodstream. Complex starches, oligosacharides, and disaccharides must be broken down into their individual monosaccharides before being absorbed in the duodenum and the upper jejunum. This digestion is facilitated by enteric enzymes, including pancreatic α-amylase and α-glucosidase, that are attached to the brush border of the intestinal cells [18]. In diabetes mellitus, control of postprandial plasma glucose levels is critical early in treatment. Inhibition of the enzymes involved in the metabolism of carbohydrates is one of the therapeutic approaches for delaying postprandial hyperglycemia. Acarbose is an antidiabetic drug that acts by delaying the digestion of carbohydrates [22]. It is a competitive inhibitor of the intestinal α-glucosidase enzyme [18]. The averted sac method used in this present study showed that ME managed to inhibit glucose absorption from the intestine, yielding a similar effect to that of acarbose. This property may have partly contributed to the antihyperglycemic activity of *S. polyanthum* leaf extract.

ME effect on muscle glucose uptake was also assessed in the current study with/without insulin and with the use of metformin as a positive control. The major effect of insulin on the muscles is to stimulate the translocation of the insulin-responsive glucose transporter, glucose transporter-4 (GLUT4), from the intracellular vesicles to the cell surface; hence, it promotes amino acid uptake, stimulates the ribosomal protein synthesis machinery, and increases the rate of glycogen synthase activity and subsequent glycogen storage, while decreasing the rate of glycogen breakdown [23,24,25]. Metformin, a biguanide, is an insulin-sensitizing agent with potent antihyperglycemic properties [26]. Due to its actions via insulin receptors and glucose transporters [27], it reduces glucose absorption in the small intestines; increases glucose uptake into the cells and its use by target tissues; decreases plasma free fatty acid concentrations; and inhibits the process of gluconeogenesis, thereby decreasing insulin resistance. The activation of AMP-dependent protein kinase (AMPPK) plays an important role in these processes [25]. Since insulin resistance contributes greatly to the metabolic syndrome and is the major cause of type 2 diabetes mellitus, treatment with insulin sensitizers may ameliorate the pathophysiological abnormalities of the metabolic syndrome [26]. Biguanides activate AMPPK to block the breakdown of fatty acids, and to inhibit hepatic gluconeogenesis and glycogenolysis [24,28]. The effect of metformin on the peripheral insulin-sensitive tissues requires the presence of insulin for a full action [26]. In the absence of added insulin, there would only be minor effects [29]. Nevertheless, the direct effect of the drug on the glucose transport system has been proven as it enhanced glucose analogue transport in a fashion independent of insulin [21,30]. In the isolated abdominal muscle model of the present study, *S. polyanthum* leaf ME enhanced glucose uptake by the muscles both with and without the presence of insulin, which was similar to the behavior of metformin. In the presence of insulin, the extract enhanced the sensitivity of insulin to increase the uptake of glucose, whereas in the absence ofinsulin, the action was probably due to an effect on the glucose transporters that directly contributed to the uptake of glucose. This finding supported the previous *in vivo* studyin the streptozotocin-induced diabetic rat model, and this mechanism contributed to the antihyperglycemic action of *S. polyanthum* leaves.

Investigation of the phytochemical compounds present in the methanolic extract of *S. polyanthum* leaves showed it to contain tannins, flavonoids, glycosides and alkaloids. Previously, it has been shown that the administration of tannin fractions from *Ficus racemosa* for 30 days significantly reversed the increase of the blood glucose levels of an STZ-induced hypercholesterolemia-associated diabetic rat model [31]. Furthermore, some flavonoids have been reported to have antihyperglycemic activity through various mechanisms of action, such as the inhibition of α-glucosidase and the increment of blood insulin levels [32,33]. Moreover, certain flavonoids, e.g., quercetin, glycoside [34] and phytol have been known to be able to regenerate pancreatic β-cells [35]. On the other hand, alkaloid-rich fractions of *Capparis decidua* showed antidiabetic potential in mice [36]. Other studies reported that alkaloids (vindoline, vindolidine, vindolicine and vindolinine) induced relatively high glucose uptake in amouse β-TC6 pancreatic cell line and mouse myoblast (skeletal muscle) C2C12 cells. Improving glucose uptake by pancreatic or muscle cells could reduce hyperglycemia in type 2 diabetes [37].

The GC-MS analysis of ME showed the presence of squalene, a triterpene that is an isoprenoid compound [38]. It belongs to the terpenoid family and has a similar structure to β-carotene [39]. It contains six isoprene units andhas been known to possess several beneficial properties [38], including an antioxidant activity [40]. Squalene has been presented in several studies as a compound that contributes to the antihyperglycemic activity of plants. n-Hexadecanoic acid, octadecanoic acid and squalene were present among the phytochemicals in *Mucuna pruriens* and *Cynodondactylon* and showed an antihyperglycemic activity on STZ-induced diabetic rats [37,41]. The hypoglycemic action of the fruit seed extract of *Syzygium cumini*, which contains triterpenes, has also been experimentally verified [16]. Squalene obtained from plants [42,43] acts as an α-glucosidase inhibitor [44] and may increase insulin secretion [45].

## 5. Conclusions

In conclusion, the antihyperglycemic effect of the methanolic extract of *S. polyanthum* leaf may be attributed to the presence of flavonoids, glycosides and squalene. The effect is probably exerted by the extra-pancreatic pathway via inhibition of intestinal glucose absorption and enhancement of glucose uptake by the muscles.
